# S1R mediates NRF2 dependent ferroptosis of renal tubular epithelial cells to promote renal fibrosis in diabetic nephropathy

**DOI:** 10.7150/ijms.104324

**Published:** 2025-01-27

**Authors:** Cheng Yuan, Fengpei Chang, Qiuyuan Zhou, Feng Chen, Xueyun Gao, Ayinigaer Yusufu, Jinhu Chen, Zejin Liao, Xiaoyan Wu, Lihua Ni

**Affiliations:** 1 Department of Oncology, Yichang Central People's Hospital and The First College of Clinical Medical Science, China Three Gorges University Yichang, Hubei, China.; 2 Tumor Prevention and Treatment Center of Three Gorges University and Cancer Research Institute of Three Gorges University Yichang, Hubei, China.; 3 Clinical Medical Research Center for Precision Diagnosis and Treatment of Lung Cancer and Management of Advanced Cancer Pain of Hubei Province.; 4 Department of Nephrology, Zhongnan Hospital of Wuhan University, Wuhan, Hubei, China.; 5 Department of Nephrology, Xijing Hospital, Fourth Military Medical University, No. 127 Chang le West Road, Xi'an, Shanxi, 710032, China.; 6 Department of Pathology, The Central Hospital of Enshi Tujia and Miao Autonomous Prefecture, Enshi, 445000, China.; 7 Department of General Practice, Zhongnan Hospital of Wuhan University, Wuhan, Hubei, 430071, China.

**Keywords:** Diabetic nephropathy, ferroptosis, sigma-1 receptor (S1R), renal fibrosis, nuclear factor erythroid 2-related factor 2 (NRF2)

## Abstract

**Rationale**: Tubulointerstitial fibrosis is a key pathological aspect of diabetic nephropathy (DN) linked to reduced kidney function. Recent research has identified varied functions of the sigma-1 receptor (S1R) in the pathological fibrosis processes of cardiac, pulmonary, and trabecular meshwork tissues. Nonetheless, the specific roles of S1R in renal fibrosis remain inadequately understood.

**Objective:** This study sought to examine the roles of S1R in the pathogenesis of diabetes-induced renal fibrosis, as well as to elucidate the underlying mechanisms involved.

**Materials and methods:** S1R expression was found in DN patients, db/db mice, and HG-treated HK-2 cells. Loss-of-function studies confirmed S1R's role in nuclear factor erythroid 2-related factor 2 (NRF2) pathway-mediated ferroptosis and renal fibrosis. Molecular docking and co-immunoprecipitation (CO-IP) analysis explored the S1R-NRF2 interaction.

**Results:** S1R was primarily found in tubular epithelial cells and was up-regulated in DN patients, db/db mice, and HG-cultured HK-2 cells. S1R inhibition alleviated ferroptosis and fibrosis in HG stimulated HK-2 cells, while knockdown of NRF2 further abolishes these protective effects by S1R inhibition. As for the further mechanism, S1R combined with NRF2 in HK-2 cells, and knockdown of S1R could increase NRF2 nuclear translocation and up-regulate the expression of phosphorylated NRF2 (p-NRF2), and finally ameliorate ferroptosis and ferroptosis-mediated renal tubular fibrosis.

Conclusions: In DN, S1R expression in the kidneys is significantly elevated. It increases p-NRF2 expression, which inhibits NRF2 nuclear translocation, promoting ferroptosis in renal tubular epithelial cells and resulting in tubular fibrosis.

## Introduction

Diabetic nephropathy (DN) is one of the most common and severe microvascular complications of diabetes and has been the leading cause of chronic kidney disease (CKD) 1. Its pathological changes include glomerular sclerosis and tubular-interstitial fibrosis (TIF), which inevitably develop into renal fibrosis. Previous research by other scholars, as well as our own team, has demonstrated that tubular ferroptosis plays a crucial role in the development of diabetic nephropathy (DN) 2-4. Specifically, mechanisms related to ferroptosis, including oxidative stress, iron overload and inflammatory response exhibit several similarities with the pathological characteristics of DN-TIF 5, 6. Moreover, inhibiting ferroptosis in tubular epithelial cells (TECs) could alleviate DN associated renal fibrosis 4. Therefore, inhibiting ferroptosis in TECs could potentially serve as a novel therapeutic target for addressing DN-TIF.

Sigma receptors (SRs) were initially discovered in the nervous system in 1976 7. Later on, they were also be found in peripheral tissues, including kidney, heart, retina and liver 8. SRs were first thought to be a subtype of opioid receptors, but ligand binding tests later revealed that their binding site is distinct 9. SRs are now recognized as an independent receptor family with two subtypes: Sigma1R (S1R) and Sigma2R (S2R). Recent studies have highlighted various roles of S1R in fibrosis affecting cardiac, lung, and trabecular meshwork tissues. Qu et al 10 propose that S1R protects against cardiac fibroblast activation by blocking the IRE1 pathway and restoring autophagic flux, making it a potential therapeutic target for cardiac fibrosis. Cao et al 11 reveal that inhibiting the S1R reduces endoplasmic reticulum stress and fibroblast changes caused by SiO_2_, offering new insights for developing silicosis treatments. Hodrea et al 12 discover that the S1R agonist fluvoxamine may offer a new glaucoma treatment by reducing F-actin rearrangement, inhibiting cell proliferation and migration caused by platelet-derived growth factor, and lowering fibrotic protein levels. However, the roles of S1R in DN-induced TIF remains unclear. Hosszu et al 13 discovered that S1R is present in proximal tubules but absent in glomeruli, and demonstrated that activating S1R protects against renal ischemia-reperfusion injury by triggering Akt-mediated nitric oxide signaling. Milardović et al 14 revealed S1R expression was highest in distal tubules and significantly increased in the kidneys of diabetic rats, with a strong co-expression alongside the proinflammatory factor TGF-β. In hepatocellular carcinoma cells, S1R prevents ferroptosis by boosting GPX4 expression and reducing iron metabolism and lipid peroxidation 15. Based on these results, we speculated that S1R might contribute to tubular damage and fibrosis in DN, but the involvement of ferroptosis remains to be elucidated.

Several studies suggest that S1R regulates ROS accumulation via NRF2, which play an important role in ferroptosis 13, 16-18. The Kelch ECH-associating protein 1 (KEAP1)-nuclear factor erythroid 2-related factor 2 (NRF2) signaling pathway is crucial for defending against internal and external stress. Under normal conditions, KEAP1 dimerizes and binds NRF2, keeping it in the cytoplasm where excess NRF2 is degraded. When cellular stress increases, KEAP1 changes shape, releasing NRF2 to move to the nucleus and activate the antioxidant response 19-21. The nuclear translocation of NRF2 constitutes a critical protective mechanism by which the body mitigates lipid peroxidation and ferroptosis 22, 23. In related research, it has been demonstrated that sulforaphane can inhibit ferroptosis in myocardial cells of diabetic mice through the upregulation of nuclear NRF2^24^. Barwick et al 25 revealed that S1R co-localizes with NRF2 in retinal photoreceptor cells.

Above all, we investigated S1R's role and mechanism in tubular ferroptosis and renal fibrosis, examining if the NRF2 pathway is involved in S1R-regulated ferroptosis, to offer new strategies for treating renal fibrosis in DN.

## Materials and Methods

### Patients

The renal tissue samples of patients with biopsy-proven DN were enrolled in the study. Control samples of healthy kidney tissue were obtained from distant portions of kidneys surgically excised because of the presence of a localized neoplasm. The study was approved by the Clinical Trial Ethical Committee, Zhongnan Hospital of Wuhan University [No. 2023052K].

### Animals

Ten C57BL/6J wild-type mice and ten db/db mice were purchased from Changzhou Cavince Laboratory Animal Co., Ltd. They were housed in well-ventilated plastic cages with a 12:12 h light-dark cycle at 22±3°C and free to food and water. After 8 weeks, all mice were anesthetized by intraperitoneal injection of pentobarbital sodium. Anesthetized mice were perfused with physiological saline about 5 minutes, then kidneys were rapidly dissected for subsequent study. All studies were in accordance with the Guide for the Care and Use of Laboratory Animals published by the National Institutes of Health. The present study was approved by the Animal Care and Ethics Committee, Zhongnan Hospital of Wuhan University [No. ZN202315].

### Histologic evaluation

Collected kidney tissues were fixed in 4% paraformaldehyde, paraffin-embedded and cut into 3μm thick slices. After xylene dewaxing and gradient ethanol hydration, slices were stained with hematoxylin and eosin (HE) and periodic acid-Schiff's (PAS) to assess renal damage such as glomerular hypertrophy, mesangial matrix expansion, tubular injury. Masson staining was used to evaluated the degree of renal fibrosis.

### Cell culture

HK-2 (human kidney proximal tubular cells) were obtained from Procell Life Science&Technology Co.,Ltd. Cells were cultured in Dulbecco's Modified Eagle Media (DMEM) containing 5.5 mmol/L glucose and 10% fetal bovine serum in 5% CO_2_ and 95% humidity at 37°C. Cells were seeded in 6-well plates at a density of 12×10^6^/peer well. When the fusion rate of cells rose to 70-80%, the media was replaced with serum-free media and cells were starved for 24h to promote cell synchronization. Subsequently, cells in control group were cultured in serum-free media containing 5.5 mmol/L glucose. Cells in HG group were cultured in serum-free media containing 30 mmol/L glucose. Cells in mannitol group were cultured in serum-free media with 24.5 mmol/L mannitol and 5.5 mmol/L glucose. Mannitol group was used to exclude a possible effect of osmolality on cells. Cells for Si-S1R group were treated with S1R transfections and cultured in 30 mmol/L glucose serum-free media for 48h. Cells for Erastin group were treated with 10 μM Erastin for 24h. Cells for Si-S1R+Si-NRF2 group were treated with S1R and NRF2 transfections and cultured in 30 mmol/L glucose serum-free media for 48h.

### SiRNA transfection

SiRNAs were purchase from Shanghai Gene Pharma Co., Ltd. Transient SiRNA transfection was performed by Lipofectamine 2000. 5ul of Lipofectamine 2000 and 200ul of Opti-MEM serum-free media were mixed and stewing 5 min.7.5ul of 20uM SiRNA and 200ul of Opti-MEM serum-free media were mixed and stewing 5 min. Subsequently, the two mixtures were mixed together and incubated for 20 min, then transferred into 6-well plate combined with 1.6 mL of Opti-MEM serum-free media. After 6h of transfection, cells were treated with HG for 48h. The silence efficiency of SiRNAs were analyzed by quantitative RT-PCR.

### RNA extraction and quantitative real-time PCR (qRT-PCR)

Total RNA of kidneys and HK-2 cells was extracted by using trizol. The concentration and purity of RNA were detected by using UV spectrophotometer. The primers sequences of each gene were listed in Table 1 and Table 2. Then RNA was reverse-transcribed into cDNA. qRT-PCR was performed on the C1000 Touch Thermocycler CFX96 Real-Time System. The relative mRNA expression was processed by the 2^-ΔΔCt^ method.

### Western blotting analysis

Total protein of kidneys and HK-2 cells was extracted by using RIPA lysis buffer and PMSF at a ratio of 99:1. The concentration of protein was analyzed by BCA protein concentration quantitative method. Samples were electrophoretically resolved on 10% or 15% polyacrylamide gels and transferred to polyvinylidene difluoride membranes. After blocking, polyvinylidene difluoride membranes were incubated with primary antibodies at a dilution ratio of 1:1000 at 4°C overnight. Primary antibodies include Sigma1R (sc-137075, Santa Cruz Biotechnology), α-SMA (sc-53142, Santa Cruz Biotechnology), Fibronectin (sc-8422, Santa Cruz Biotechnology), NRF2 (sc-365949, Santa Cruz Biotechnology), ACSL4 (sc-365240, Santa Cruz Biotechnology), GPX4 (sc-166570, Santa Cruz Biotechnology), KEAP1 (10503-2-AP, Proteintech), GAPDH (GB11002, Servicebio), β-tubulin (GB11017, Servicebio). After washing 3 times, they were incubated with goat anti-rabbit or anti-mouse secondary antibody at a dilution ratio of 1:5000 for 1h at room temperature. After repeated washing, the specific band was visualized using the ECL Imaging System, and the optical density of each band was measured using the ImageJ software.

### Immunohistochemistry (IHC) and immunofluorescence (IF) analysis

The sections were put in a 60°C oven overnight. After xylene dewaxing, gradient ethanol hydration and heat-induced epitope retrieval, the sections were incubated in primary anti-S1R antibody (1:75, sc-137075, Santa Cruz Biotechnology), anti-ACSL4 antibody (1:75, sc-365240, Santa Cruz Biotechnology) or anti-α-SMA antibody (1:75, sc-53142, Santa Cruz Biotechnology) at 4°C overnight. The next day, the sections were incubated with secondary antibody.

For NRF2 immunofluorescence staining, HK-2 cells were incubated overnight at 4°C with the respective primary and secondary antibodies. Sections or cells were then stained with 4,6-diamidino-2-phenylindole (DAPI, Wuhan bioqiandu technology CO., China, B0011) dye and antifading medium. Images were taken using a fluorescence microscope.

### Iron, GSH and MDA assay

The collected cells were homogenized by ultrasonic disrupter. After centrifugation for 10 minutes, the supernatant was collected to measure the iron, GSH and MDA concentrations in cells. They were measured by Iron Assay Kit (Solarbio, Beijing, China), Micro Reduced Glutathione GSH Assay Kit (Beyotime, Shanghai, China) and Micro Malondialdehyde Assay Kit (Solarbio, Beijing, China), respectively. Subsequently, microplate reader was used to detect the OD value of cells in each well.

### Co-immunoprecipitation

Cells were lysed by IP Lysis buffer for 10 min. The lysate was then incubated with 5μl S1R (sc-137075, Santa Cruz Biotechnology), NRF2 (sc-365949, Santa Cruz Biotechnology), and mouse monoclonal IgG antibody (sc2025, Santa Cruz Biotechnology) at 4 °C overnight. 5ul of Protein A and Protein G magnetic beads were mixed in the lysate and incubated at 4 °C overnight to form the immune complex. After washing three times, western blotting analysis was used for further study.

### Molecular docking

For molecular docking, the 3D crystal structures of NRF2 and S1R were downloaded from the RCSB Protein database (https://www.rcsb.org/). The AutoDock tools were operated for dock and analyze protein complexes. Pymol were conducted for draw the interaction of NRF2 and S1R according to the docking results.

### Bodipy fluorescence staining

To evaluate lipid peroxidation, HK-2 cells were stained with BODIPY 581/591 C11, incubated at 37 °C for 30 minutes in the dark, washed with PBS, and stained with DAPI. Images were analyzed using a fluorescence microscope.

### CCK8 assay

Cell viability assay was conducted using the Cell Counting Kit-8 (CCK-8, Abbkine, BMU106-CN). Cells (1 × 10^4 per well) were seeded in 96-well plates. Post-treatment, the medium was replaced with 100 μl of CCK-8 and incubated for 1-2 hours, followed by measuring absorbance at 450 nm.

### Statistical analysis

All data were analyzed by using GraphPad Prism 8.0 software. Differences between two groups were used Student's t-test. For multiple-group comparison, one-way ANOVA analysis was performed. P values < 0.05 were considered significant.

## Results

### The renal expression of S1R was increased in DN patients, db/db mice and HG cultured HK-2 cells

**Figure 1:**
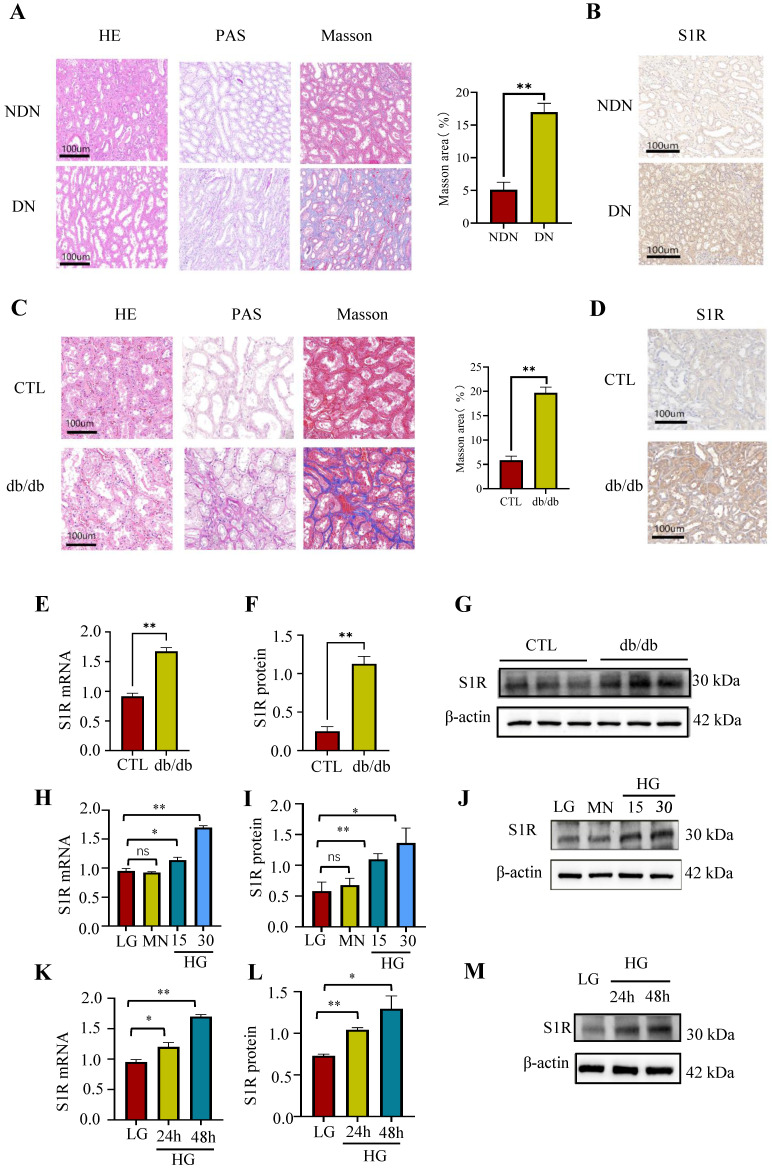
The expression of S1R was increased in DN. (A) (C) HE, PAS, and Masson staining of renal sections in DN patients (upper) and db/db mice (lower). (B) (D) lmmunohistochemical staining for S1R in DN patients (upper) and db/db mice (lower); (E-G) The mRNA and protein expression of S1R in db/db mice; (H-M) The mRNA and protein expression levels of S1R were analyzed in cultured HK-2 cells subjected to varying durations and concentrations of glucose exposure. Each experiment was repeated at least three times independently, values were means ± SD. DN means diabetic nephropathy. NDN means non-DN, CTL means the normal control animals. MN means mannitol. *p < 0.05, **p < 0.01, ns means no significance.

To assess the renal expression of S1R in individuals with DN, renal biopsy specimens were obtained from these patients. Histopathological analysis indicated the presence of glomerulosclerosis, while tubular atrophy was demonstrated using HE and PAS staining. Additionally, interstitial fibrosis was identified through Masson's trichrome staining in the DN patient samples (Figure 1A). Subsequently, immunohistochemistry was performed to explore the expression of S1R in DN. As shown in Figure 1b, the renal expression of S1R was increased in DN, and S1R prominently localized in tubular epithelial cells (TECs) but not in glomeruli (Figure 1B).

Next, investigations about the renal expression of S1R was performed in db/db mice. As well known, tubulointerstitial and glomerular fibrosis is one of the distinct features for pathological diagnosis of type 2 DN. The db/db mice showed renal TEC shedding, brush border loss, severe tubular vacuolization, tubular swelling, and interstitial fibrosis, unlike the normal controls, as revealed by histopathological examination (Figure 1C). The immunohistochemistry of renal tissues showed increased S1R expression specifically in TECs in db/db mice, compared to the normal controls (Figure 1D). Accordingly, the mRNA and protein expression levels of S1R were significantly increased in db/db mice (Figure 1E-G).

As the above discussed, renal S1R are primarily located in TECs in both DN patients and animal models. Consequently, HK-2 cells under high glucose (HG) conditions were utilized for *in vitro* studies. As anticipated, the expression levels of S1R protein and mRNA exhibited an increase in a manner dependent on both time and concentration under high-glucose conditions (Figure 1H-M). Taken together, the expression of renal S1R was elevated in DN and predominantly localized in TECs. However, the function of S1R in the context of DN requires further comprehensive investigation.

### Ferroptosis was observed along with renal fibrosis in DN patients, db/db mice

**Figure 2:**
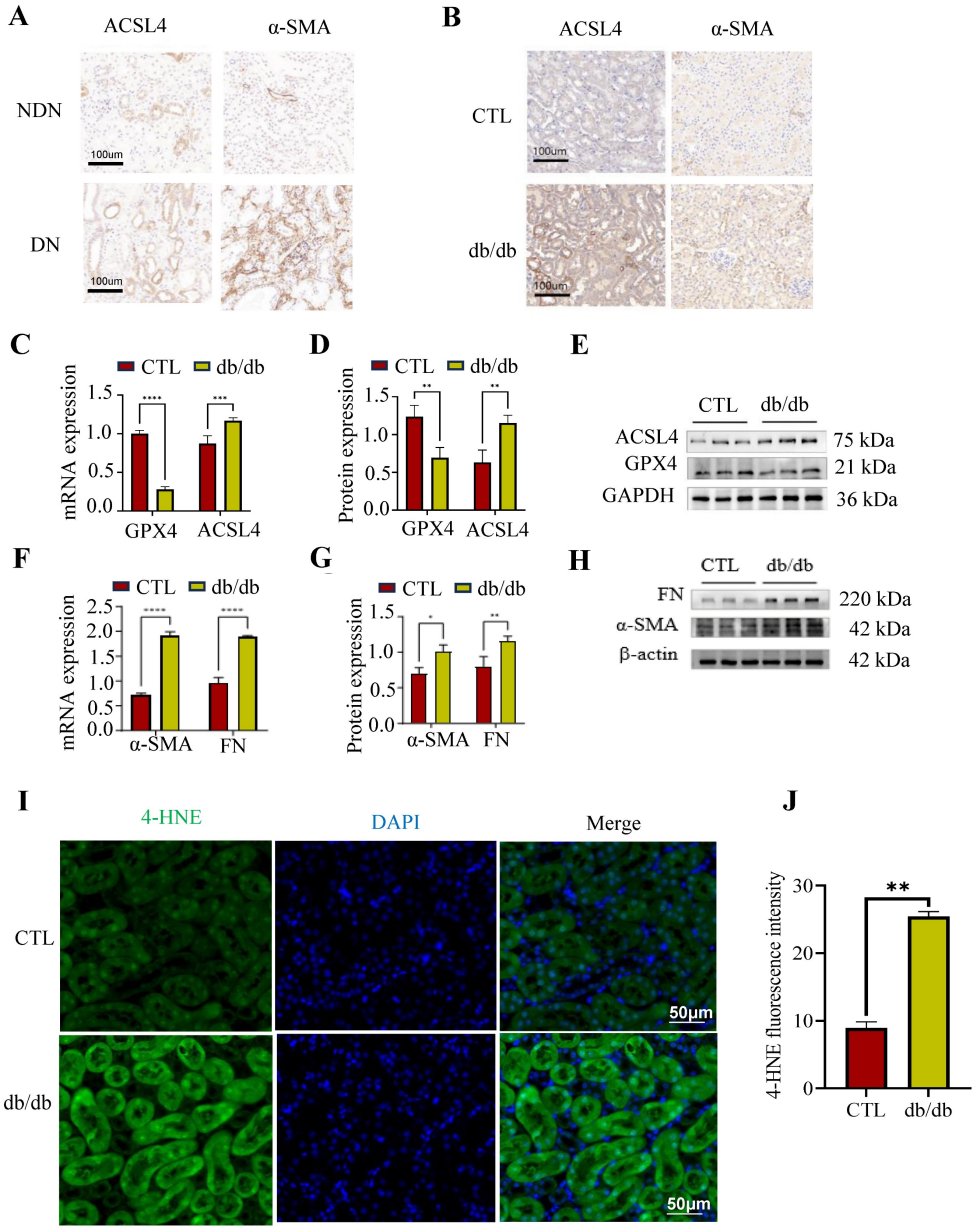
Tubular ferroptosis was activated along with renal fibrosis in DN patients and animals. (A-B) Immunohistochemical staining for ACSL4 and α-SMA in DN patients (left) and db/db mice (right). (C-H) The mRNA and protein expression of ferroptosis-related makers (ACSL4 and GPX4) and fibrosis-related makers (FN and α-SMA) in db/db mice. (I-J) The 4-HNE staining observed in db/db mice is depicted on the left, accompanied by the corresponding quantitative analysis on the right. Each experiment was repeated at least three times independently. All results were presented as the mean ± SD. *p < 0.05, **p < 0.01, ***P < 0.001, and ****P < 0.0001.

Previous studies had proved that renal tubular ferroptosis could enhance renal tubular injury and renal fibrosis in DN^26^. Here, we detected ferroptosis- and fibrosis-related markers in DN patients and db/db mice. GPX4 is key in combating oxidative stress, ACSL4 aids lipid biosynthesis linked to ferroptosis, and Fibronectin (FN) along with α-smooth muscle actin (α-SMA) are related to extracellular matrix (ECM) deposition. The renal results of immunohistochemistry showed that the ACSL4 and α-SMA were increased in DN patients and db/db mice, compared to the normal controls (Figure 2A, B). These findings were in concordance with the PCR and Western blotting results obtained from db/db mice (Figure 2C-H). The lipid ROS were increased in the proximal tubules in db/db mice, as determined by an IF against 4-HNE (indicator of oxidation production) (Figure 2I-J).

These data suggest that ferroptosis was observed along with renal fibrosis in DN patients and db/db mice.

### Ferroptosis was observed in HG cultured HK-2 cells

**Figure 3:**
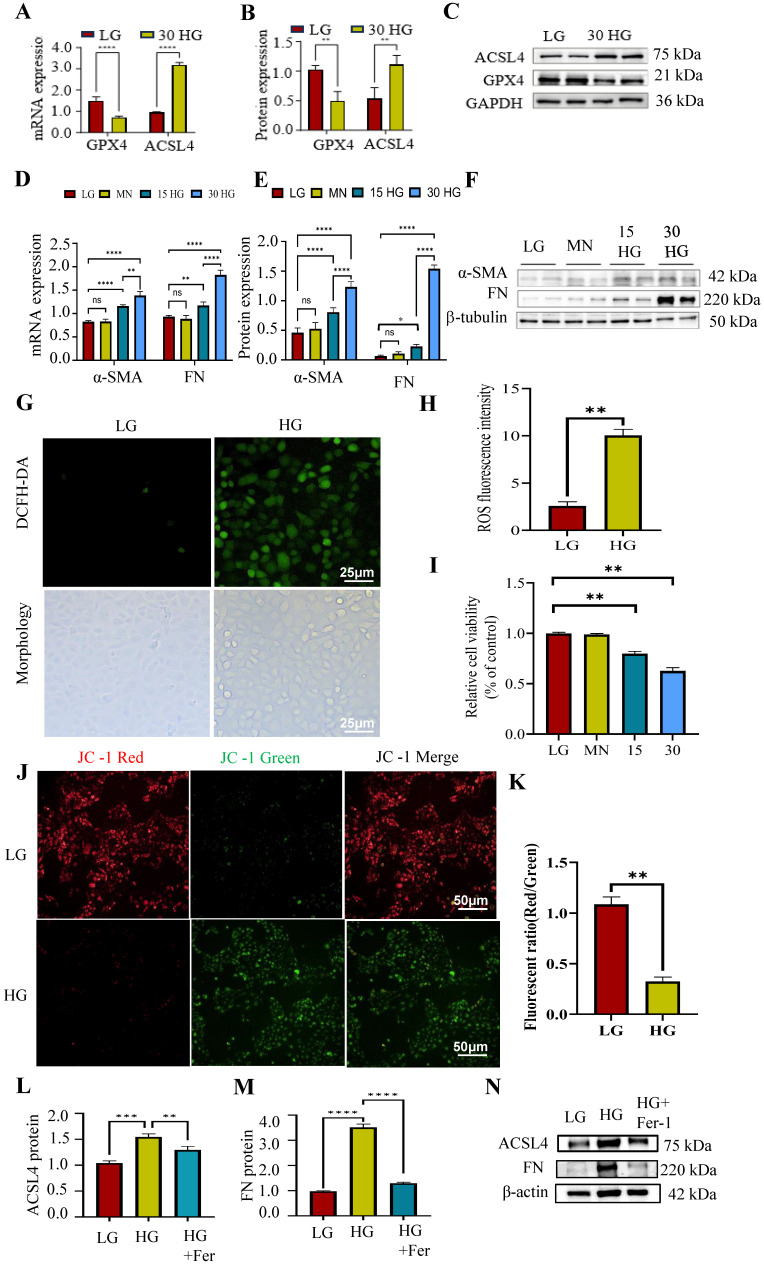
Tubular ferroptosis was activated along with renal fibrosis in HG-cultured HK2 cells. (A-F) The mRNA and protein expression of ACSL4, GPX4, α-SMA and FN was detected under HG treatment. (G-H) The ROS generation. (I)CCK8 assay. (J-K) JC-1 stain. (L-N) The mRNA and protein expression of ACSL4, and FN was detected under HG and Fer treatment. Each experiment was repeated at least three times independently MN means mannitol. All results were presented as the mean ± SD. *p < 0.05, **p < 0.01, ***P < 0.001, and ****P < 0.0001. ns means no significance.

Then the relation between ferroptosis and renal fibrosis *in vitro* were investigated, and the HG incubated HK-2 cells were adopted in this part. As shown in Figure 3A-F, the mRNA and protein expression of pro-fibrotic markers (α-SMA, FN) and pro-ferroptosis marker (ACSL4) were increased while anti-ferroptosis marker (GPX4) was decreased under the elevated HG stimulation. Besides, there was no significance between mannitol group and the normal controls which excluded the effects of high osmotic pressure on HK-2 cells.

Reactive oxygen species (ROS) facilitate lipid peroxidation, a critical mechanism in the process of ferroptosis. As shown in Figure 3G-H, we observed alter morphology and enhanced ROS generation after HG incubation in HK-2 cells. Besides, the relative cell viability was decreased under HG condition (Figure 3I), which means HG stimulation could induce cell death.

Prior research has demonstrated that excessive generation of ROS can induce morphological alterations in mitochondria, thereby contributing to cellular apoptosis 27. Maintaining mitochondrial membrane potential is vital for cell survival and can be monitored using JC-1 staining, which shows a fluorescence shift from red to green if the potential decreases. As shown in Figure 3J-K, the cells displayed increased green fluorescence and decreased red fluorescence under HG incubation. This suggested HG stimulation could alter the mitochondrial membrane potential.

Subsequently, to investigate the potential involvement of ferroptosis in HG-induced renal fibrosis, we cultured HK-2 cells in the presence of HG and Ferrostatin-1 (Fer-1), a widely recognized ferroptosis inhibitor. Fer-1 treatment reduced the elevated ACSL4 and FN expression caused by HG incubation (Figure 3L-N). These data indicated that HG could induce fibrosis through enhancing tubular ferroptosis.

The data indicates that ferroptosis significantly contributes to HG-induced renal fibrosis, and understanding its mechanism could offer insights for targeting renal fibrosis in DN.

### S1R mitigated ferroptosis, and its inhibition suppressed ferroptosis and renal fibrosis *in vitro*

**Figure 4:**
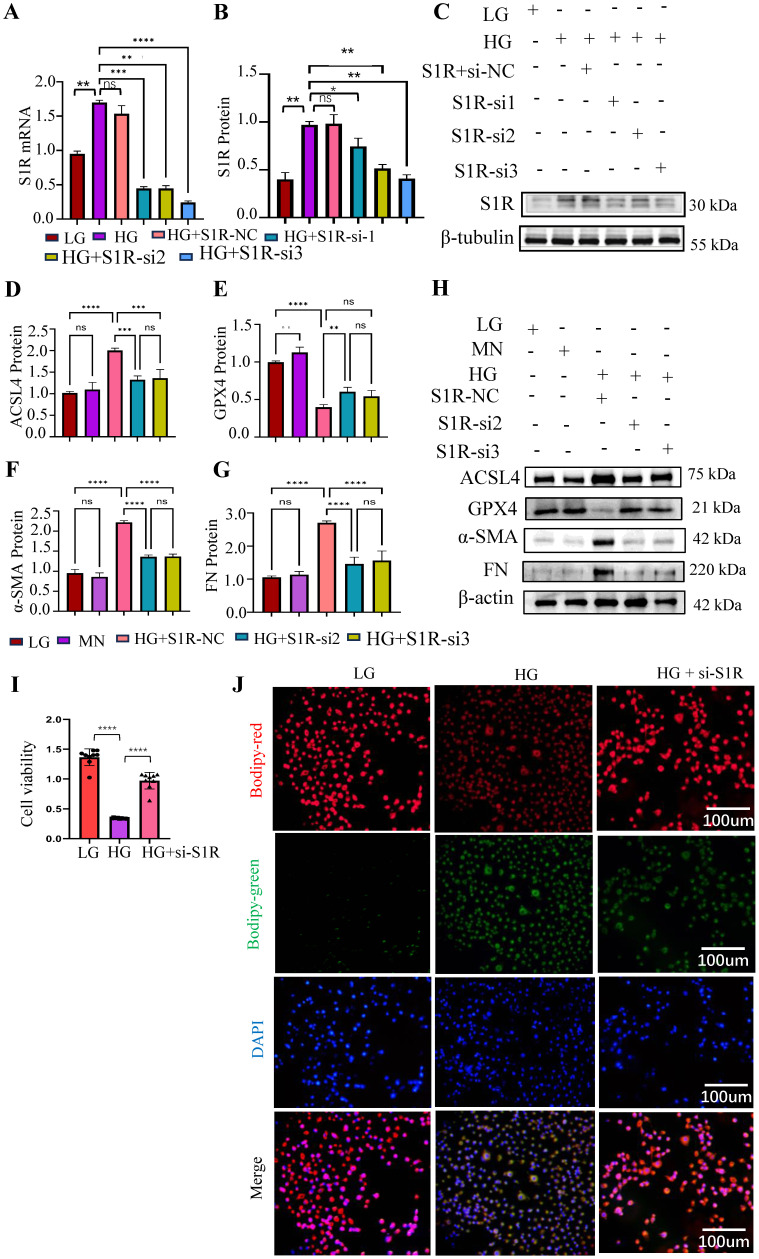
S1R inhibition suppressed tubular ferroptosis and renal fibrosis. Small interfering RNAs (siRNA) targeting S1R was transfected with HK-2 cells. (A-C) S1R knockout efficiency was validated by PCR and Western blotting. And S1R-si2 and si3 achieved the best knock effects among the 3 siRNAs. (D-H) HK-2 cells were transfected with S1R-si2 and si3, the protein expression of ferroptosis and fibrosis-related makers were detected by western blotting. (I) Cell viability was measured by CCK8 in HK-2 cells subjected to HG and S1R blockage. n=10 for each group (J) BODIPY detected lipid peroxidation in HK-2 cells treated with HG, with or without S1R inhibition. Each experiment was repeated at least three times independently. MN means mannitol. All results were presented as the mean ± SD. *p < 0.05, **p < 0.01, ***P < 0.001, and ****P < 0.0001. ns means no significance.

To explore the effects of S1R in ferroptosis and renal fibrosis, the small interfering RNA (siRNA) targeting S1R were synthesized to knockdown the expression of S1R. As shown in Figure 4A-C, S1R-si2 and S1R-si3 significantly reduced S1R expression, with S1R-si3 exhibiting the most pronounced effect, making it the preferred choice for subsequent investigations. The related indicators of ferroptosis (ACSL and GPX4) and fibrosis (α-SMA and FN) were determined by Western blotting. As shown in Figure 4D-H, HG stimulation could increase the expression of ACSL, FN, α-SMA, and decrease the expression of GPX4, which could be alleviated by S1R inhibition. When cells undergo ferroptosis, their viability is significantly reduced. As shown in Figure 4I, HG stimulation could decrease the cell viability in HK2 cells, which were reversed after S1R inhibition. During ferroptosis, lipid peroxides accumulate within the cell, and these peroxides can react with the BODIPY dye. As shown in Figure 4J, it was found that the fluorescence signals in the HG group was elevated compared with the LG group, but S1R inhibition impeded HG-induced production of lipid peroxides.

**Figure 5:**
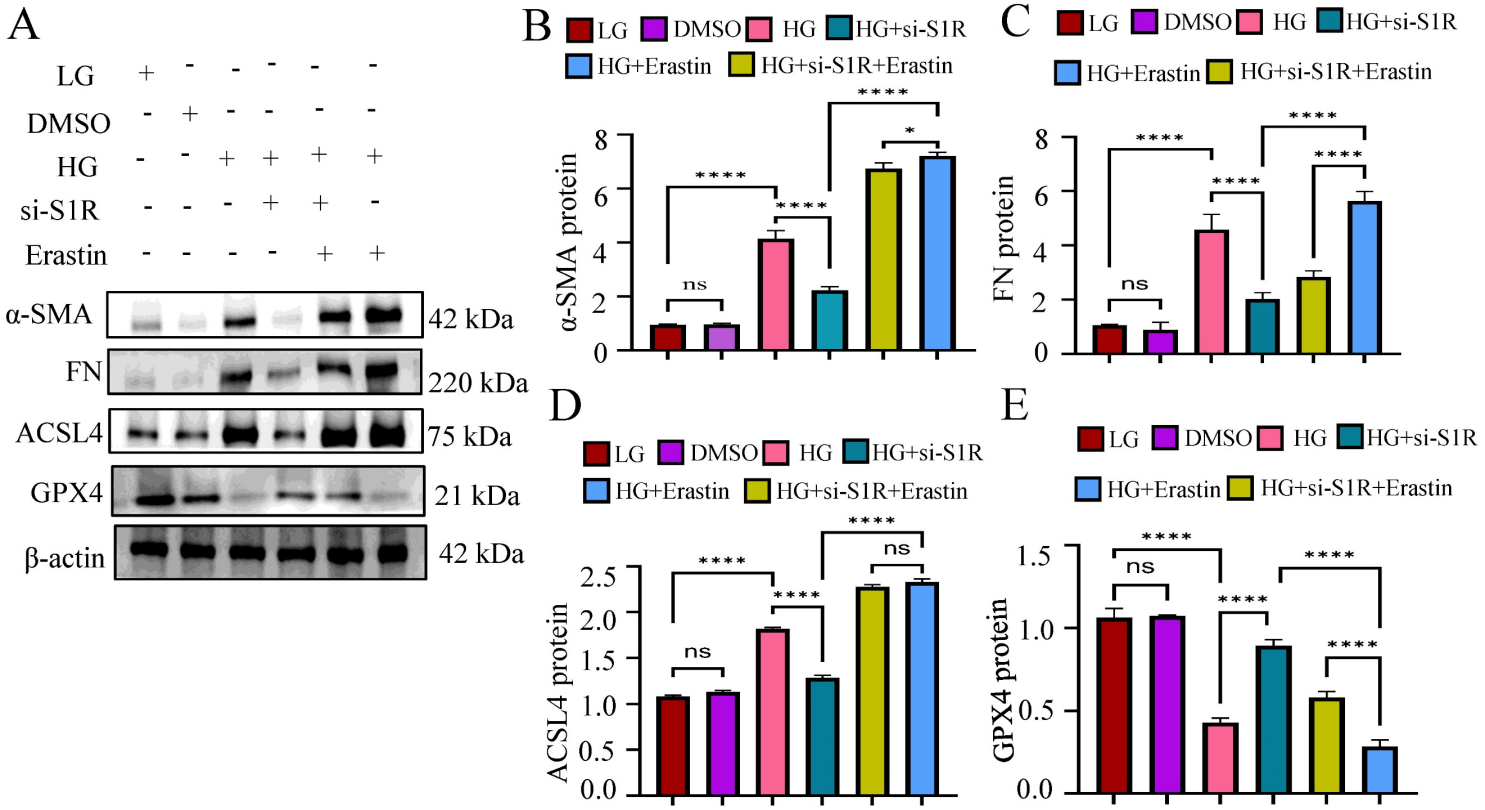
Erastin counteracted the beneficial effects of S1R inhibition on renal fibrosis. S1R-si3 was selected, and transfected with HK-2 cells. Erastin, a well known ferroptosis inducer was applied. The protein expression of ferroptosis (ACSL4 and GPX4) and fibrosis-related makers (FN and α-SMA) were detected by western blotting. Each experiment was repeated at least three times independently. All results were presented as the mean ± SD. *p < 0.05, **p < 0.01, ***P < 0.001, and ****P < 0.0001. ns means no significance.

To verified whether the amelioration of S1R inhibition on renal fibrosis was achieved by inhibiting ferroptosis, we further evaluated the effects of Erastin (A widely used inducer for ferroptosis) on renal fibrosis *in vitro*. We examined the related indicators of ferroptosis (ACSL and GPX4) and fibrosis (α-SMA and FN) under HG incubation, S1R-siRNA transfection and Erastin stimulation. As shown in Figure 5, S1R inhibition improved renal fibrosis and ferroptosis caused by HG. Interestingly, after adding Erastin, these anti-ferroptosis and anti-fibrosis changes were alleviated, indicating that the improvement of S1R inhibition on renal fibrosis could be achieved by preventing ferroptosis.

These data demonstrated that S1R was involved in tubular ferroptosis induced by HG, and S1R inhibition could decrease ferroptosis and renal fibrosis.

### The inhibition of NRF2 eliminated the effects of S1R blockage on HG-induced ferroptosis

**Figure 6:**
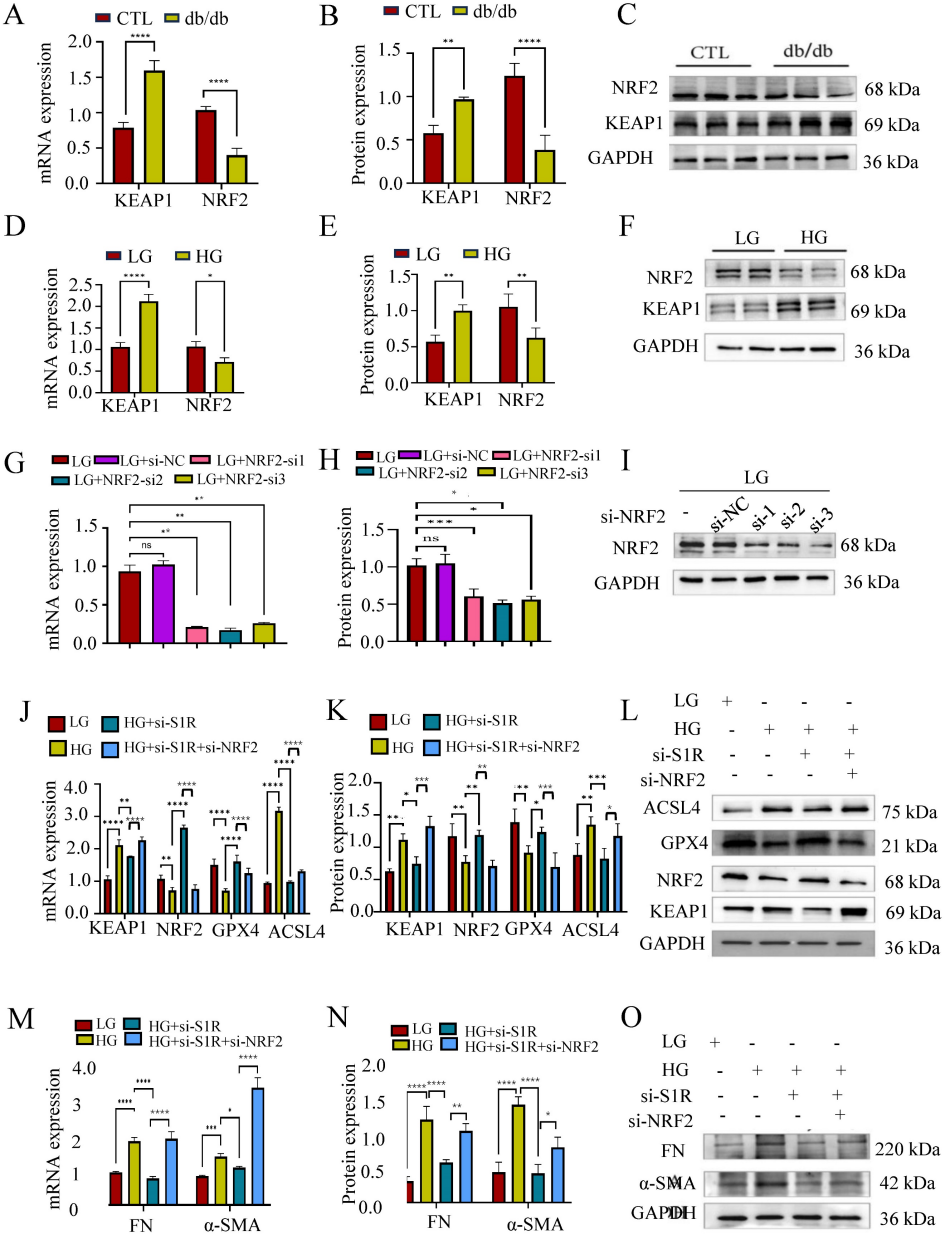
Inhibiting NRF2 negated the impact of S1R blockage on HG-induced ferroptosis. (A-F) The mRNA and protein expression of NRF2 was detected in db/db mice and HG-treated HK-2 cells. (G-I) Small interfering RNAs (siRNA) targeting NRF2 was transfected with HK-2 cells, and the NRF2 knockout efficiency was validated by PCR and Western blotting. And NRF-si2 achieved the best knock effects among the 3 siRNAs. (J-O) HK-2 cells were transfected with S1R-siRNA and NRF2-siRNA to perform the loss-function study. The mRNA and protein expression of ferroptosis markers, NRF2 pathway, and fibrosis markers were detected. Each experiment was repeated at least three times independently. All results were presented as the mean ± SD. *p < 0.05, **p < 0.01, ***P < 0.001, and ****P < 0.0001.

How S1R mediate tubular ferroptosis in renal fibrosis is still unclear? NRF2 is a transcription factor that typically resides in the cytoplasm bound by KEAP1, responsible for resisting oxidative stress and plays a crucial role in iron metabolism and ferroptosis. To evaluate whether the KEAP1-NRF2 pathway was involved in the protective effect of S1R inhibition on HG-induced ferroptosis and renal fibrosis, we examine the renal expression of NRF2 and KEAP1 in db/db mice and HG incubated HK-2 cells by Western blotting. As expected, the renal KEAP1-NRF2 pathway was suppressed in db/db mice and HG-incubated HK2 cells (Figure 6A-F).

Next, we checked the roles of NRF2 pathway in S1R regulated ferroptosis. HK-2 cells were transfected with siRNA targeting S1R and NRF2 to knockdown the expression of S1R and NRF2. As shown in Figure 6G-I, NRF2-si2 significantly decreased NRF2 expression, which obtain the best blocking effects among the three NRF2-siRNAs. S1R blocking could attenuate HG-induced ferroptosis and renal fibrosis as indicated by Western blotting. While these changes could be abolished by NRF2 inhibition (Figure 6J-O).

**Figure 7:**
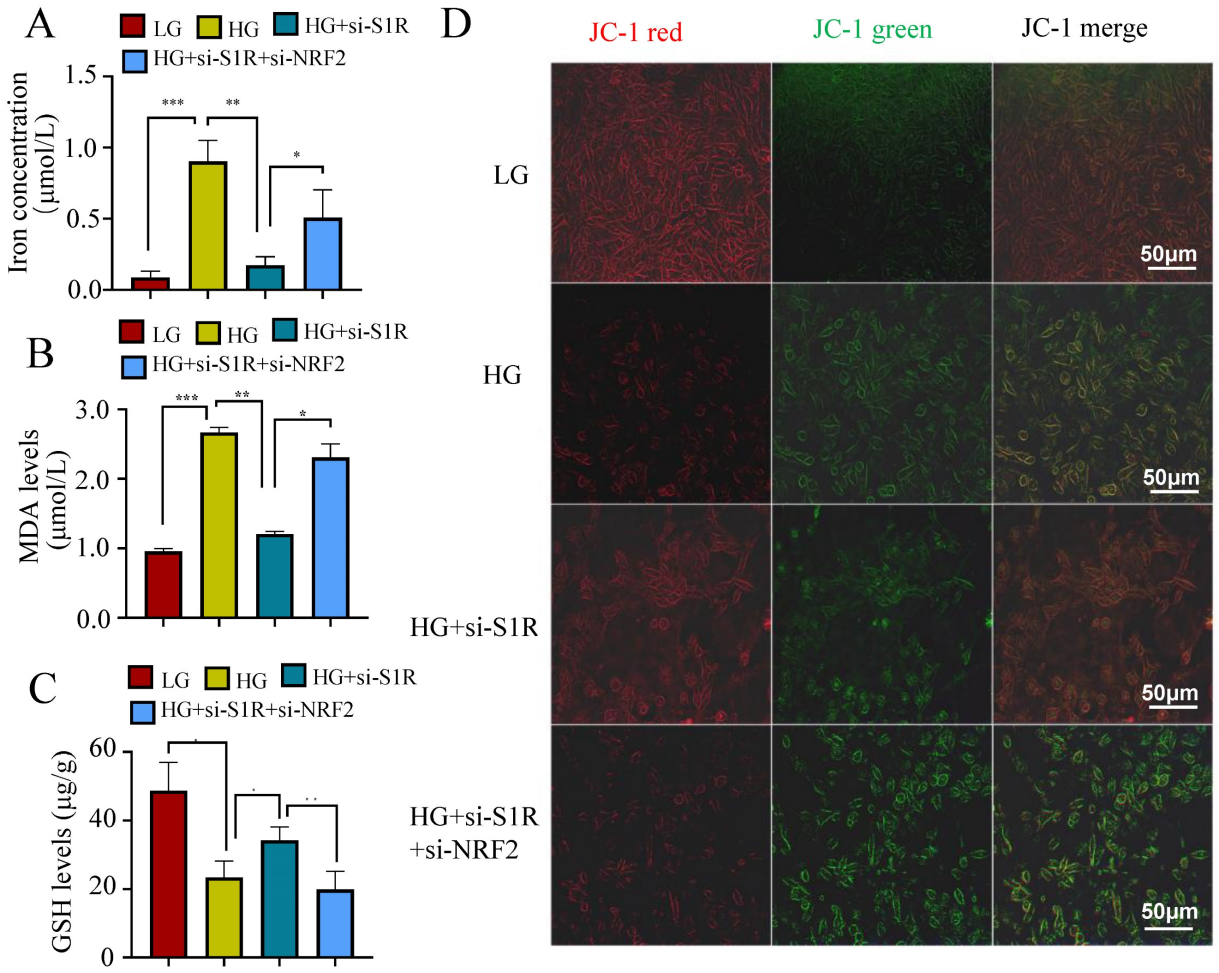
S1R blockage iron concentration, MDA levels and GSH levels through NRF2 regulation. (A) iron levels; (B) MDA contents; (C) GSH levels; (D) JC-1 staining. All results were presented as the mean ± SD. Each experiment was repeated at least three times independently, *p < 0.05, **p < 0.01, ***P < 0.001, and ****P < 0.0001.

Other indicators connected to ferroptosis were also performed to evaluate the roles of S1R in NRF2 pathway. As we all known, ferroptosis is an iron-dependent type of cell death defined by lipid peroxidation. Excessive iron or iron overload can induce oxidative stress damage and ferroptosis. GSH could detoxify lipid peroxidation and inhibit ferroptosis. The detection about iron and GSH levels were conducted. As shown in Figure 7A-B, a significantly higher iron content and lower GSH contents were observed in HG incubated HK-2 cells when compared with the controls. S1R inhibition attenuate these changes, which could be further reversed by NRF2 blocking. MDA, a finally products of lipid peroxidation, was strongly increased in HG group. S1R inhibition could decrease the MDA levels, which could be further reversed by NRF2 blocking (Figure 7C).

Whether S1R and NRF2 expression influenced the mitochondrial membrane potential were detected by the fluorescent dye JC-1. The S1R-siRNA-transfected cells displayed decreased green fluorescence and increased red fluorescence under HG incubation, and NRF2 inhibition could restore the effects of S1R blocking (Figure 7D). This suggest that changes in S1R and NRF2 expression could alter the mitochondrial membrane potential.

Taken together, NRF2 pathway was involved in S1R mediated ferroptosis, while the exactly mechanism between S1R and NRF2 pathway still need further study.

### S1R targeted NRF2 and induced its nuclear translocation in HK-2 cells

**Figure 8:**
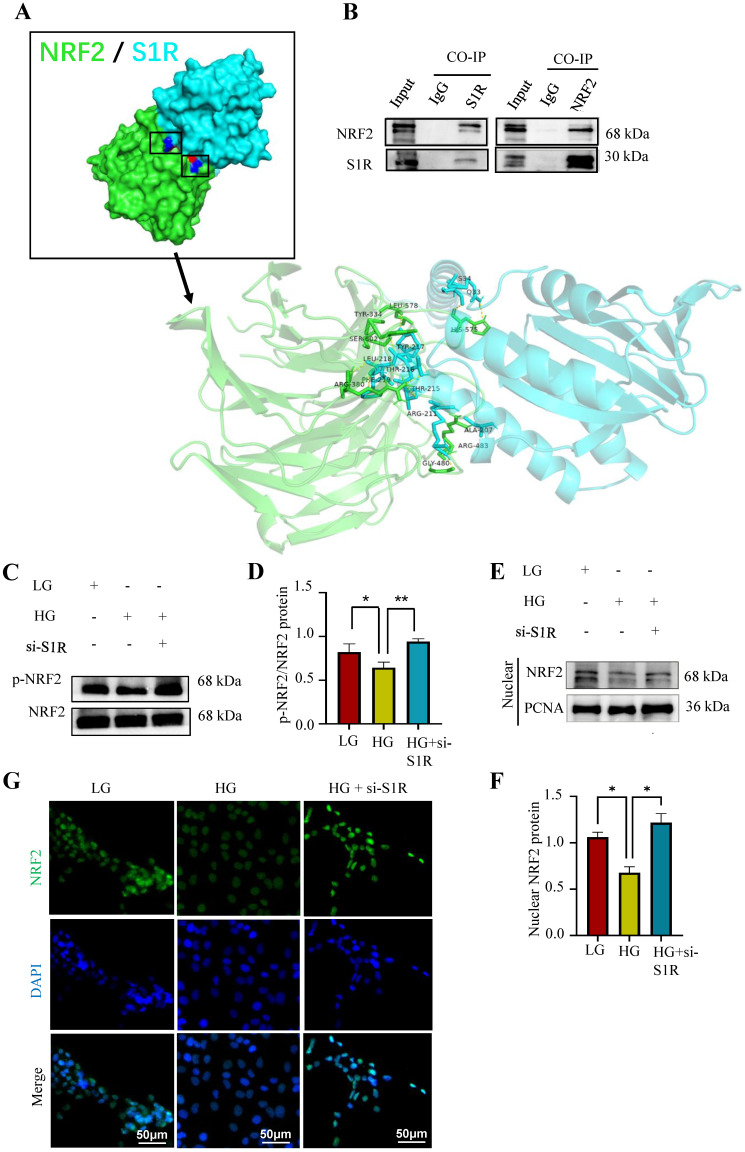
S1R directly targeted NRF2 and induced its nuclear translocation in HK-2 cells. (A) Molecular docking was performed to study interaction of NRF2 and S1R. (B) The interaction of NRF2 and S1R was further confirmed by co-immunoprecipitation. (C-D) Expression of p-NRF2 and NRF2 was detected by Western blotting. Quantification of p-NRF2/NRF2 was determined. (E-F) Expression of nuclear NRF2 was detected by Western blotting. Quantification of nuclear levels by normalizing to PCNA. (G) Detection of NRF2 translocation to the nuclei was investigated by Immunofluorescence staining in HK-2 cells incubated with HG in the presence or absence of S1R inhibition. Cells were stained with NRF2 (green) and DAPI (blue). Each experiment was repeated at least three times independently. All results were presented as the mean ± SD. *p < 0.05, **p < 0.01.

To explore how S1R regulated NRF2 pathway, Autodock vina and Pymol for molecular docking and interaction analysis were performed in Figure 8A, we conducted protein docking between NRF2 and S1R. The docking results suggest that the huge possibility of NRF2 bind to S1R. Then Co-IP methods was used to further confirm the interaction between S1R and NRF2. Western blotting analysis detected NRF2 in the S1R-bead complex from the IP procedure, as well as S1R itself. And S1R was also detected in the NRF2-bead complex (Figure 8B-C). These data revealed that S1R could bind with NRF2 protein.

The activation of NRF2 pathway depends on the dephosphorylated levels of NRF2, then facilitation of NRF2 nuclear translocation and activating the transcription of genes that have antioxidative effects. It is important to delineate how S1R down-regulate the NRF2 pathway. Firstly, as shown by Western blotting analysis, we found the proteins levels of p-NRF2/NFR2 was decreased in HG-exposed HK-2 cells and further increased in si-S1R transfection group (Figure 8D). Then we extracted nuclear protein for the follow-up study in HK-2 cells, and found that the nuclear expression of NRF2 was decreased in HG group but increased in S1R inhibition group. These results together suggested that HG stimulation can inhibit NRF2 translocate to the nucleus, and S1R knockdown can increase NRF2 nuclear translocation (Figure 8E, F). Indeed, the immunofluorescence staining images showed that S1R inhibition promoting NRF2 from the nucleus to cytoplasm in HK-2 cells treated with HG (Figure 8G).

The above data confirmed that conder HG condition, S1R could bind to NRF2, decreases phosphorylation levels of NRF2 and prevents the nuclear translocation NRF2.

## Discussion

With the development of DN research, the role of renal tubulointerstitial lesions and interstitial fibrosis in the progression of DN had widely concerned 28-30. However, the underlying mechanism still need in-depth research. In the present study, we observed a significant upregulation of renal S1R expression in DN. S1R interacts with cytoplasmic NRF2, inhibiting its nuclear translocation. This inhibition facilitates ferroptosis in renal tubular epithelial cells, ultimately contributing to tubular fibrosis. This study is the first to demonstrate that S1R induce the progression of ferroptosis in DN-TIF through the NRF2 pathway.

It has been proposed that S1R participated in a variety of disorders including inflammation, depression, drug addiction, and neuropathic pain 31, 32. As for renal, the roles of S1R are differed. In the animal model of renal ischemia-reperfusion injury, up-regulation of S1R significantly improves postischaemic survival and renal function by enhancing the heat shock response 13. In the animal model of streptozotocin (STZ)-induced diabetes, renal S1R was significantly increased, and co-located with TGF-β, a typically proinflammatory and pro-fibrotic factors 14. However, the role of S1R in DN remain largely unclear. In our study, we observed an upregulation of renal S1R expression in patients with DN, db/db mice, and HG-incubated HK-2 cells, with a predominant localization in TECs. Furthermore, inhibition of S1R markedly reduced HG-induced renal fibrosis in HK-2 cells. These findings suggest that S1R plays a protective role against pro-fibrotic effects in the context of DN.

A deeper understanding of DN's pathological processes is crucial to explore S1R's mechanism in DN-TIF. In our study, we observed a pronounced enhancement of ferroptosis and fibrosis, along with inactivation of the NRF2 pathway, in db/db mice. These findings were further corroborated through western blot analysis in HG-stimulated HK-2 cells. It is well established that NRF2 serves as a crucial transcription factor in maintaining intracellular redox homeostasis and acts as a primary regulator of lipid peroxidation 33, 34. Then, we hypothesized that S1R induces tubular ferroptosis via the NRF2 pathway, ultimately resulting in DN-TIF.

Ferroptosis is a kind of regulated cell death characterized by iron-dependent accumulation of lipid peroxides. In recent years, accumulating evidences have demonstrated that ferroptosis plays an important role in the development of acute kidney injury (AKI) 35, renal cell carcinoma 36 and polycystic kidney 37, and relatively little concrete evidence to support ferroptotic contribution to the development of DN. Wang et al. demonstrated that the activation of the vitamin D receptor (VDR) inhibits ferroptosis in TECs associated with DN through the modulation of the NRF2/HO-1 signaling pathway 38. In our previously study 2, we found that enhanced ferroptosis was associated with the progression of DN, and targeting tubular ferroptosis could recover the renal damage. Our study shows that tubular ferroptosis triggers fibrosis in HG conditions, with S1R promoting DN-TIF by inducing tubular ferroptosis. Key findings include: HG stimulation causes ferroptosis and renal fibrosis in HK-2 cells, reversible with the ferroptosis inhibitor Fer-1. Additionally, S1R inhibition prevents HG-induced renal fibrosis, which can be reinstated by the ferroptosis inducer Erastin. Above all, we initially demonstrated that S1R's pro-fibrotic effect in DN-TIF may involve inhibiting tubular ferroptosis, though the precise mechanism requires further investigation.

To clarify S1R's role in tubular ferroptosis and renal fibrosis, we examined the NRF2 pathway in db/db mice and HG-stimulated HK-2 cells. Generally, NRF2 is the core transcription factor for cells to resist oxidative stress and plays a key role in iron metabolism 22, 39. Numerous proteins and enzymes implicated in lipid peroxidation and ferroptosis, including GPX4 and ACSL4, are downstream targets of the NRF2 signaling pathway 22. The activity of NRF2 is rigidly regulated by an inhibitor named KEAP1, a member of the BTB-Kelch family that is rich in cysteine residues 40. A previous study 16 credibly shows that S1R controls oxidative stress response by influencing the KEAP1-NRF2 pathway in retinal Müller glial cells. Our study found that inhibiting S1R may reduce tubular ferroptosis and renal fibrosis in DN, effects negated by blocking NRF2. Co-immunoprecipitation showed NRF2 and S1R interact. Additionally, S1R knockdown enhances NRF2 nuclear translocation under high glucose conditions. Its nuclear translocation is a key step in activating the transcription of antioxidant response element (ARE)-driven genes, which are essential for cellular protection against oxidative stress and damage. Upon oxidative stress or other stimuli, KEAP1 undergoes conformational changes or inactivation, allowing NRF2 to escape degradation and translocate into the nucleus 41, 42. Post-translational modifications, such as phosphorylation, can also enhance NRF2 stability and facilitate its nuclear translocation 43, 44. These data together supported that NRF2 mediated ferroptosis accounts for S1R induced DN-TIF.

In conclusion, our study indicated that S1R mediated ferroptosis was involved in DN-TIF. Mechanistically, in the condition of DN, the S1R is increased, which interacts with cytoplasmic NRF2, decrease p-NRF2 and inhibit the translocation of NRF2 into the nucleus. Consequently, this process induces ferroptosis in renal tubular epithelial cells, ultimately contributing to tubular fibrosis. S1R or tubular ferroptosis may represent a promising therapeutic candidate for the treatment of DN-TIF.

## Figures and Tables

**Table 1:** Primer sequences

Target gene	Forward primer (5'-3')	Reverse primer (5'-3')
human KEAP1	CAACCGACAACCAAGACCC	ATAAGCAACACCACCACCTC
mouse KEAP1	ATAAGCAACACCACCACCTC	TGCCCCTGTGGTCAAAGTG
human GPX4	CTGGACGAGGGGAGGAG	CGACGAGCTGAGTGTAGTTT
mouse GPX4	GATGGAGCCCATTCCTGAACC	CCCTGTACTTATCCAGGCAGA
human ACSL4	ACTGGCCGACCTAAGGGAG	GCCAAAGGCAAGTAGCCAATA
mouse ACSL4	CTCACCATTATATTGCTGCCTGT	TCTCTTTGCCATAGCGTTTTTCT
human NRF2	CGCAGACATTCCCGTTTGTA	AGTTTGGCTTCTGGACTTGG
mouse NRF2	TCTTGGAGTAAGTCGAGAAGTGT	GTTGAAACTGAGCGAAAAAGGC
human β-actin	CATGTACGTTGCTATCCAGGC	CTCCTTAATGTCACGCACGAT
mouse β-actin	GGCTGTATTCCCCTCCATCG	CCAGTTGGTAACAATGCCATGT
human-Sigma1R	CGAAGAGATAGCGCAGTTGG	TCCACGATCAGACGAGAGAAG
mouse Sigma1R	CATTCGGGACGATACTGGGC	CCTGGGTAGAAGACCTCACTTTT
human fibronectin	CGGTGGCTGTCAGTCAAAG	AAACCTCGGCTTCCTCCATAA
mouse fibronectin	ATGTGGACCCCTCCTGATAGT	GCCCAGTGATTTCAGCAAAGG
human α-SMA	AAAAGACAGCTACGTGGGTGA	GCCATGTTCTATCGGGTACTTC
mouse α-SMA	GTCCCAGACATCAGGGAGTAA	CGGATACTTCAGCGTCAGGA

**Table 2:** Primer sequences for si-RNA

Target gene	Forward primer (5'-3')	Reverse primer (5'-3')
NRF2-1	GGUUGAGACUACCAUGGUUTT	AACCAUGGUAGUCUCAACCTT
NRF2-2	CCAGAACACUCAGUGGAAUTT	AUUCCACUGAGUGUUCUGGTT
NRF2-3	GCCUGUAAGUCCUGGUCAUTT	AUGACCAGGACUUACAGGCTT
Sigma1R-1	GGCUGGGUACGCAGAGCUUTT	AAGCUCUGCGUACCCAGCCTT
Sigma1R-2	CCGAGUAUGUGCUGCUCUUTT	AAGAGCAGCACAUACUCGGTT
Sigma1R-3	GCUGAGAUCUCGGAUACCATT	UGGUAUCCGAGAUCUCAGCTT
